# Temporal and spatial pattern, sources, and main controlling factors of odor compounds in Qiandaohu Reservoir

**DOI:** 10.1007/s10661-025-14505-5

**Published:** 2025-08-20

**Authors:** Yi Chi, Zhanpu Tang, Yingjun Zhu, Lingjia Wang, Tianli Chen, Ping He, Yuxin Chen, Tong Sun, Siying Cai, Weijun zhang

**Affiliations:** 1 Hangzhou Ecological and Environmental Monitoring Center of Zhejiang Province, Hangzhou, 310003 Zhejiang China; 2 Quality Testing Center for Architectural Engineering of Zhejiang Province Co. Ltd, Hangzhou, 310012 Zhejiang China; 3 School of Water Resources and Environmental Engineering, East China University of Technology, Shanghai, 200237 China; 4 School of Environmental Studies, China University of Geosciences, Wuhan, 430074 Hubei China

**Keywords:** Odor compounds, Geosmin, 2-Methylisoborneol, Algae

## Abstract

The ubiquitous presence of algal-derived odors in large lakes and reservoirs constitutes a critical environmental concern. Particularly during the high-temperature summer months, localized regions of Qiandaohu Reservoir have exhibited markedly elevated concentrations of odor compounds. To investigate the intricate relationships among odor compounds, algae communities, and environmental factors in Qiandaohu Reservoir, and to forestall the impact of odor compounds on the quality of drinking water, this research implemented a systematic monthly survey to monitor several types of odor compounds, algal communities, physicochemical factors, and associated synthetases. The result demonstrated that the odor issue in Qiandaohu Reservoir is not yet severe. Among the detected compounds, dimethyl sulfide, dimethyldisulfide, and β-cyclocitral were all below the odor threshold, while dimethyl trisulfide, 2-methylisoborneol, geosmin, and β-ionone exceeded the odor threshold in some samples. The odor compounds are jointly influenced by abiotic environmental conditions and algae, presenting conspicuous horizontal, vertical, and seasonal distribution disparities. Notably, total nitrogen, total phosphorus, and the TN/TP ratio exert a substantial influence. Algae play a pivotal role in contributing to these odor compounds, warranting particular attention to the composition and dynamic alterations of the algal community during the summer and autumn seasons. This research comprehensively investigates the spatiotemporal patterns, sources, and controlling factors of odor compounds in a large drinking water source reservoir with relatively lower trophic status, providing new insights into odor management in such ecosystems.

**Supplementary Information:**

The online version contains supplementary material available at 10.1007/s10661-025-14505-5.

## Introduction

Intensified anthropogenic activities in recent decades have substantially increased nutrient inputs into aquatic systems, particularly nitrogen and phosphorus, driving eutrophication and promoting harmful algal blooms (Harke et al., 2016). This nutrient-driven proliferation often leads to the production of taste-and-odor compounds, a critical water quality challenge particularly prevalent in lentic environments like reservoirs and lakes worldwide (Beaulieu et al., 2019; Guan et al., 2025). Odor compounds in aquatic systems exhibit extremely low sensory thresholds, with humans detecting strong odors like geosmin (GEO) and 2-methylisoborneol (2-MIB) at concentrations as low as ng/L levels (Ma et al., 2014). This hypersensitivity causes significant aversion even at trace concentrations, making precise detection methodologies critical for effective monitoring. Recent advances in analytical techniques, particularly gas chromatography coupled with chemical and sensory analysis (GC–Olfactometry), have revolutionized nuisance odor evaluation in environmental matrices (Hayes et al., 2023). These approaches not only identify compounds at perceptually relevant concentrations but also establish causal links between chemical signatures and sensory impacts. Recurrent odor events have disrupted water supplies globally, including notable incidents at Taihu Lake, Wuxi (2007, affecting > 2 million people) (Yang et al., 2008), Tianjin’s Binhai New Area (2016) (Qiao et al., 2021), and in drinking water for Shanghai (Huangpu River), Beijing (Miyun Reservoir), and along the Yellow River (Su et al., 2015; Wang et al., 2025). These incidents underscore the imperative for comprehensive strategies to address odor presence in drinking water sources and emphasize the importance of proactive prevention and mitigation measures.

Algal metabolism, particularly by cyanobacteria, is a primary source of taste-and-odor compounds in eutrophic reservoirs (Juttner & Watson, 2007; Zamyadi et al., 2012). Over 40 algal species are implicated, notably filamentous cyanobacteria like *Anabaena spp.*, *Planktothrix spp.*, and *Oscillatoria spp.* (Lu et al., 2019; Xu et al., 2019). Among the most pervasive and problematic compounds are 2-MIB and GEO, responsible for earthy/musty odors and mandated for monitoring in drinking water systems (Bristow et al., 2019). These compounds are mandated for testing in both finished water and distribution system water (Wang et al., 2015). Other significant taste-and-odor compounds include sulfurous compounds (dimethyl sulfide (DMS), dimethyl disulfide (DMDS), dimethyl trisulfide (DMTS)) associated with rotten/decaying odors, and carotenoid derivatives like β-cyclocitral and β-ionone. Wang et al. (2019) analyzed raw and treated water samples from 56 drinking water treatment plants across 31 cities in China, revealing that raw river water exhibited a higher detection rate (38.5%) of rotten/decaying odor compounds, whereas lakes and reservoirs had a higher prevalence (50.0%) of musty odor compounds. Notably, β-cyclocitral and β-ionone are well-established degradation products of β-carotene. Zhang et al. (2013) identified a strong positive correlation (*R*^2^ = 0.96) between β-cyclocitral and β-carotene during the growth phase of *Microcystis spp.*, with β-carotene tending to degrade into β-ionone under the influence of filamentous fungi.

Within this global and national context, Qiandaohu Reservoir (Chun’an County, Zhejiang Province) stands as a critical large lake-type reservoir in East China (Yu et al., 2010). It fulfills multiple roles (e.g., power generation, flood control, tourism, aquaculture, navigation), but its significance as a strategic drinking water source for the Yangtze River Delta, particularly Hangzhou and Jiaxing via the water diversion project (operational since 2019), has dramatically increased (Wu et al., 2012). However, like many reservoirs facing eutrophication pressures, Qiandaohu has experienced sporadic odor episodes in specific areas and periods following the diversion project’s full operation, posing a direct threat to its vital “large water tank” function. The reservoir’s unique hydrology and ecology, influenced by the diversion, may create specific niches for odor-producing organisms.

Therefore, given the global challenge of reservoir taste-and-odor compounds driven by nutrients and algae, and the heightened vulnerability of Qiandaohu as a primary water supply, this study aims to: (1) conduct comprehensive spatiotemporal monitoring of nutrients, algal communities (focusing on known odor-producers), odor compounds (2-MIB, GEO, DMS, DMDS, DMTS, β-cyclocitral, β-ionone), and key odor synthase genes; (2) identify the primary biological (algal) and environmental (nutrient) sources of odors within Qiandaohu; (3) analyze the spatiotemporal dynamics of these factors; and (4) elucidate the interrelationships between nutrients, algal communities, and odor production. This research will provide critical insights for targeted management strategies to mitigate odor risks and safeguard the quality and safety of Qiandaohu Reservoir’s water supply, addressing a challenge of both local urgency and global relevance.

## Materials and methods

### Research area and sampling scheme

Qiandaohu Reservoir is located between 29° 11′ N and 30° 02′ N, and 118° 34′ E and 119° 15′ E. Taking into comprehensive consideration factors such as the distribution pattern of the lake in Qiandaohu Reservoir, the water intake points for drinking water, the monitoring sections under national and provincial control, and the areas where the water source protection zones are located, a total of 17 sampling sites were set up across the entire Qiandaohu Reservoir area. Monthly stratified sampling monitoring was conducted from May 2022 to April 2024, with three independent samples collected at each site during each sampling campaign. Each collected water sample was subsequently analyzed in triplicate for all target parameters. The specific information of all sampling sites and stratified sampling of lake water were shown in Text S1 and Text S2, respectively.

The collected water samples were filtered using GF/C glass fiber filters (Whatman, UK) to separate particulate matter. The filtrate was collected in brown glass bottles for the determination of dissolved odor compounds. Meanwhile, particulate-bound odor compounds were extracted through filter membrane backwashing with methanol. All collected samples were stored at − 18 °C. In addition, phytoplankton specimens were collected through vertical towing using a No. 25 phytoplankton net. Quantitative samples were obtained by preserving 1 L of the water sample with 1.5% Lugol’s solution immediately after collection. These samples were subsequently filtered through 5 μm polycarbonate membrane filters to concentrate algae cells. The treated filter membrane was frozen in a refrigerator at − 20 °C for later fluorescence quantitative PCR analysis.

### Determination indicators and methods

#### Physicochemical indicator determination methods

Physicochemical indicators such as water temperature, pH, and dissolved oxygen (DO) were measured on-site using a YSI multi-parameter water quality analyzer. Transparency (SD) was measured on-site using a Secchi disk. The remaining physicochemical indicators were taken back to the laboratory for analysis. Total nitrogen (TN) and dissolved total nitrogen (DTN) in the water body were determined by alkaline potassium persulfate digestion-ultraviolet spectrophotometry (Zhang et al., 2023). Total phosphorus (TP) and dissolved total phosphorus (DTP) were determined by the ammonium molybdate spectrophotometric method (Yang et al., 2024). Nitrate nitrogen (NO_3_-N) was determined by ion chromatography. Nitrite nitrogen (NO_2_-N) was determined by the N-(1-naphthyl) ethylenediamine dihydrochloride colorimetric method (Pfaff, 1993). The permanganate index (COD _Mn_) was determined by the acid potassium permanganate titration method. Total organic carbon (TOC) was determined by the combustion oxidation-nondispersive infrared absorption method. Chlorophyll a (Chl.a) was determined by the spectrophotometric method after being extracted with 90% acetone in the dark at 4 °C for 24 h (Fu et al., 2023).

#### Algae identification and analysis

The water samples used for phytoplankton identification were fixed on-site with 1% Lugol’s reagent, and then left to stand and settle indoors for 48 h. The excess supernatant was removed by siphoning, and the samples were concentrated to 30 mL after sedimentation. When conducting a quantitative analysis of phytoplankton, the concentrated samples were shaken thoroughly. 0.1 mL of the sample was aspirated and dropped onto the phytoplankton counting frame. Observations were made using a microscope (Olympus CX23) at 10 × 40 magnification. The identification and counting of phytoplankton species were mainly carried out according to the “Atlas of Common Freshwater Phytoplankton in China” and the 0.1 mL counting frame-microscope counting method.

### Real-time fluorescence quantitative PCR absolute quantitative analysis

Absolute qPCR quantification of total Microcystis (16S rDNA), toxigenic Microcystis (mcyB), total cyanobacteria (cyanobacterial 16S rRNA gene), and odor-producing algae (2-MIB/GEO synthetase genes) was performed using primer sets listed in Table S1. Reactions were run in triplicate on a Bio-Rad CFX96 Touch™ Real-Time PCR Detection System (Bio-Rad Laboratories, USA) with the following 15 µL mixture: 7.5 µL 2 × AceQ qPCR SYBR Green Master Mix (Vazyme, China; Cat# Q111-02), 0.7 µL each forward/reverse primer (10 µM), 1 µL template gDNA (extracted using FastDNA Spin Kit for Soil, MP Biomedicals), and 5.8 µL nuclease-free water. Thermal cycling conditions comprised: 95 °C for 5 min; 45 cycles of 95 °C for 15 s and 55 °C for 15 s (fluorescence acquisition); followed by melt curve analysis (65 °C → 95 °C, increment 0.5 °C per 5 s) to confirm amplification specificity (Kim & Park, 2025). Absolute gene copy numbers were calculated from plasmid DNA standard curves (linearized pMD19-T vectors, Takara Bio) using CFX Maestro™ 2.3 Software (Bio-Rad) and normalized to copies per liter of original water sample.

### Determination methods for particulate and dissolved odor compounds

The enrichment of odor compounds was performed using a No.7 trap (OI Analytical Instruments, USA), and the filler was TENAX. Subsequently, the concentration of odor compounds was analyzed using a gas chromatography-mass spectrometry (GCMS-QP2010 Plus, Shimadzu, Japan) equipped with an HP-5MSUI capillary column (30 m × 0.25 mm × 0.25 μm, Agilent, USA). Helium served as the carrier gas, with an injection temperature of 270 °C, a column flow rate of 1 mL/min, and a split ratio of 10:1. The temperature program started at 50 °C (held for 2 min), increased to 15 0 °C at 10 °C/min, then to 220 °C at 5 °C/min (held for 10 min). Mass spectrometry was conducted with an ion source temperature of 200 °C, an interface temperature of 250 °C, a solvent cut time of 1.69 min, and electron impact ionization at 70 eV.

#### Sample preparation and introduction

Water samples were filtered through 0.45-μm GF/F membranes to separate particulate and dissolved phases. For dissolved odor compounds, the filtrate was enriched using solid-phase microextraction (SPME) with a TENAX-filled No.7 trap (OI Analytical Instruments, USA). For particulate-bound compounds, the filters were subjected to solvent extraction (dichloromethane:methanol, 2:1 v/v) followed by nitrogen blow-down concentration. All extracts were introduced into the GC–MS system via a programmable temperature vaporization (PTV) injector in solvent vent mode.

#### GC–MS analysis

Odor compounds were analyzed using gas chromatography-mass spectrometry (GCMS-QP2010 Plus, Shimadzu, Japan) equipped with an HP-5MSUI capillary column (30 m × 0.25 mm × 0.25 μm, Agilent, USA). Helium carrier gas flow: 1 mL/min constant flow mode. Injection: PTV injector at 270 °C (split ratio 10:1; vent pressure 5 psi, vent time 0.5 min). Oven program: 50 °C (hold 2 min) → 10 °C/min → 150 °C → 5 °C/min → 220 °C (hold 10 min). MS conditions: Electron impact ionization (70 eV); ion source 200 °C; interface 250 °C; solvent delay 1.69 min.

### Data statistics and analysis

Taxonomic Dominance and Functional Group Dominance

1 $$Y=\frac{ni}{N}\times fi$$

where *ni* is the biomass of the *i*-th dominant species or dominant functional group; *N* is the total biomass of all dominant species or dominant functional groups; *fi* is the frequency of occurrence of the *i*-th dominant species or dominant functional group; *Y* ≥ 0.2 indicates a dominant species or dominant functional group.

Shannon–Wiener Diversity Index (H), Margalef Richness Index (D), Pielous Evenness Index (e), Functional Group Diversity Shannon–Wiener Diversity Index (H′), Margalef Richness Index (D′), Pielous Evenness Index (e′):

2 $$H\left({H}{\prime}\right)=-\sum \left(\frac{Ni}{N}\right)\times \text{ln}\left(\frac{Ni}{N}\right)$$

3 $$D\left({D}{\prime}\right)=\frac{\left(S-1\right)}{\text{ln}N}$$

4 $$e\left({e}{\prime}\right)=\frac{H}{\text{ln}S}$$

where *Ni* is the total cell density of the ith phytoplankton or the ith functional group; *N* is the total cell density of all phytoplankton; *S* is the number of phytoplankton species or the number of functional groups.

## Results and discussion

### Seasonal variation of dissolved and particulate odor compounds in Qiandaohu Reservoir

Based on the spatiotemporal dynamics of seven odor compounds in Qiandaohu Reservoir, the primary contributors to odor exhibit distinct seasonal shifts. Specifically, summer and autumn were the main periods for the odor of Qiandaohu Reservoir. The dissolved DMS and DMDS were the primary odor compounds in Qiandaohu Reservoir in summer. Their peak values all occurred during the high-temperature summer months of July, August, and September, while the concentrations in other months were relatively lower. With the gradual cooling into the autumn, the main contributors to the stink of the lake began to change to 2-MIB, GEO, and DMTS. Although DMTS exhibits low theoretical solubility (3.6 mg/L at 20 °C, EPI Suite v4.1), its detection in the “dissolved” (operationally defined as < 0.45-μm filtered) phase is attributed to three synergistic mechanisms: (1) DOM-enhanced partitioning: humic acids increase DMTS apparent solubility via hydrophobic binding (log K_DOM_ = 2.54), enabling aqueous-phase partitioning (Howard, 1991); (2) Colloid-facilitated transport: algal-derived nanoparticles (< 200 nm) form stable complexes with DMTS, allowing passage through 0.45-μm filters (Chow et al., 2021); (3) Ultra-trace analytical capability: SPME-GC/MS quantification reliably detects field concentrations (0.10 ± 17.94 ng/L). Especially for 2-MIB, it exhibited relatively high concentrations during the high-temperature months of August, September, and October, with lower values observed in other months. In winter, due to the sudden drop in lake surface temperature, the slow biological metabolism of algae leads to the concentration of almost all odor compounds reaching a minimum, such as 2-MIB in January 2024 with 0.17 ng/L. Finally, in addition to the concentration peak of dissolved β-cycloidal and its degradation products (β-ionone) observed in August 2023, we also observed their relatively high values in April 2024. Therefore, the unique seasonal characteristics of β-cycloidal and β-ionone can contribute to the odor of Qiandaohu Reservoir in spring.

The concentration changes of different particulate odor compounds throughout the year are shown in Table 1. Except for β-ionone, the concentrations of other particulate odor compounds were lower than those of dissolved odor compounds, indicating that odor compounds tend to dissolve in the water environment and emit unpleasant odors. Among them, the annual average content of particulate β-Cyclocitral, particulate β-ionone, and particulate 2-MIB was the highest, and their concentrations are 2.35 ng/L, 1.45 ng/L, and 0.197 ng/L, respectively. In addition, consistent with the dissolved odorous substances, the corresponding particulate odorous compounds also have obvious seasonal characteristics. It is worth noting that the concentration peaks of all particulate odor compounds appear almost in summer and autumn, and reach the lowest in January of the following year. In July 2023, particulate DMDS and DMTS were considered to be the main contributors to the stench of suspended solids on the lake, as their peak concentrations were observed. In August, September, and October, the types of particulate odor compounds on suspended matter gradually become more abundant, including DMS, 2-MIB, GEO, β-cyclocitral, and β-ionone. For example, particulate 2-MIB exhibited relatively high concentrations during the high-temperature months of August, September, and October each year, with lower values observed in other months, and the lowest in January 2024, which was consistent with the seasonal variation in the concentration of dissolved 2-MIB.

**Table 1:** Seasonal variation of odor compounds in Qiandaohu Reservoir

Odor compounds	Dissolved concentration variations (ng/L)	Annual average of dissolved concentrations (ng/L)	Particulate concentration variations (ng/L)	Annual average of particulate concentrations (ng/L)	Odor threshold (ng/L)
DMS	0.77–7.66	2.96	0.019–0.1	0.10	2000
DMDS	0.10–4.01	0.8	0.003–3.8	0.56	4000
DMTS	0.10–17.94	1.64	0.002–0.7	0.12	10
2-MIB	0.17–32.85	4.98	0.001–1.65	0.197	10
GEO	0.06–3.4	0.7	0.007–0.33	0.092	4
β-cyclocitral	0.67–7.9	3.29	0.28–9.7	2.35	500
β-ionone	0.096–11.53	1.3	0.08–6.46	1.45	7

Consistent with previous studies, the odors from the lake are usually seasonal and closely related to algal growth. For example, Hu et al. (2017) noted during sampling that the East Lake emitted a slight foul smell from June to September, which significantly diminished or became undetectable in autumn and winter. Considering the key role of elevated water temperature in accelerating the metabolism of algae, it is necessary to pay extra attention to the eutrophication of Qiandaohu Reservoir in summer and autumn to avoid increasing the escape of odorous compounds, because we found that in the high temperature season, the concentration of odor compounds in some places will exceed the threshold, especially 2-MIB and DMTS.

### Spatial distribution characteristics of odor compounds in Qiandaohu Reservoir

#### Spatial distribution characteristics of DMS, DMDS, and DMTS

**Fig. 1:**
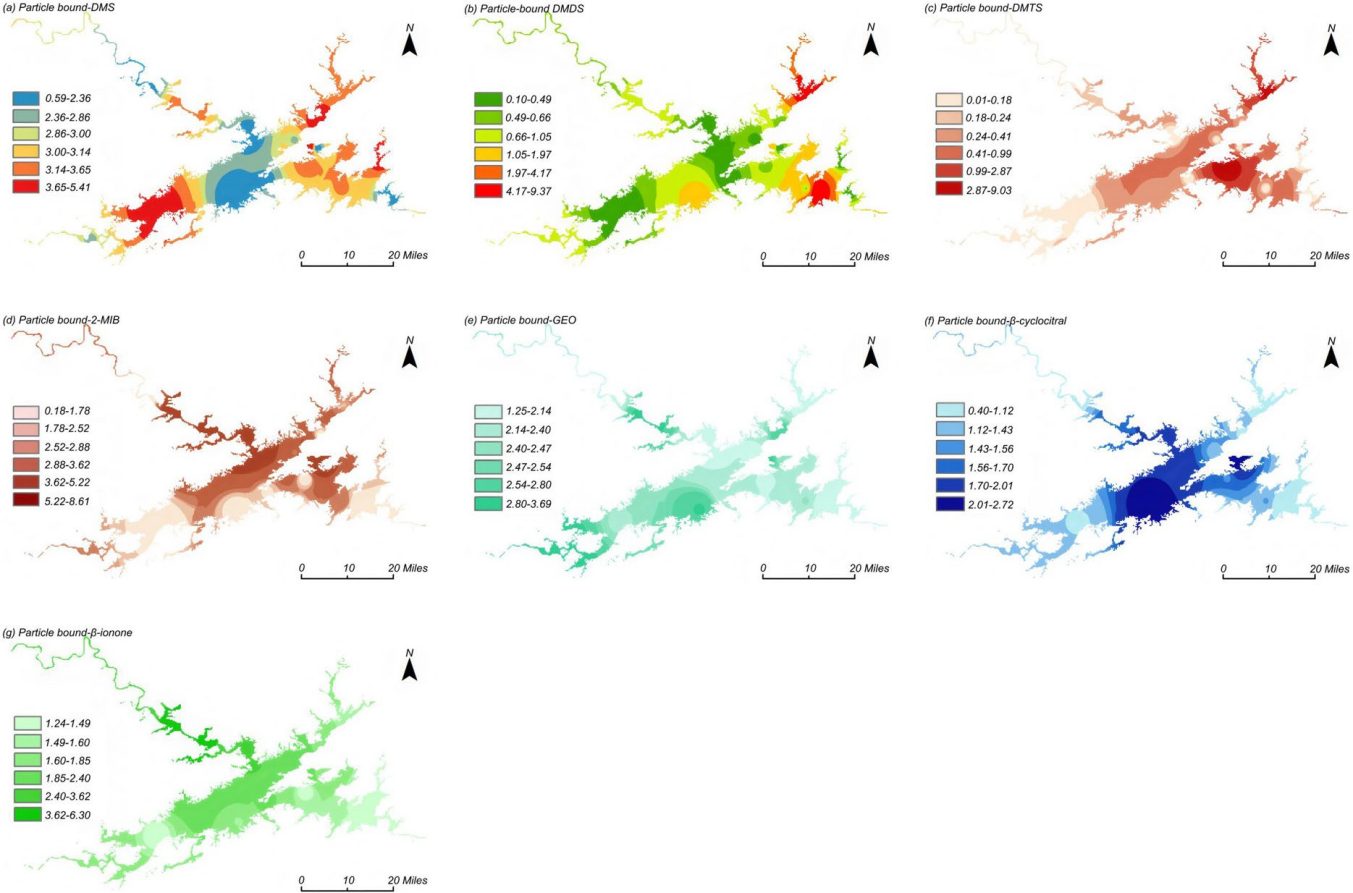
Spatial distribution characteristics of particulate odor compounds in Qiandaohu Reservoir, DMS (**a**); DMDS (**b**); DMTS (**c**); 2-MIB (**d**); GEO (**e**); β-cyclocitral (**f**); β-ionone (g) (ng/L)

**Fig. 2:**
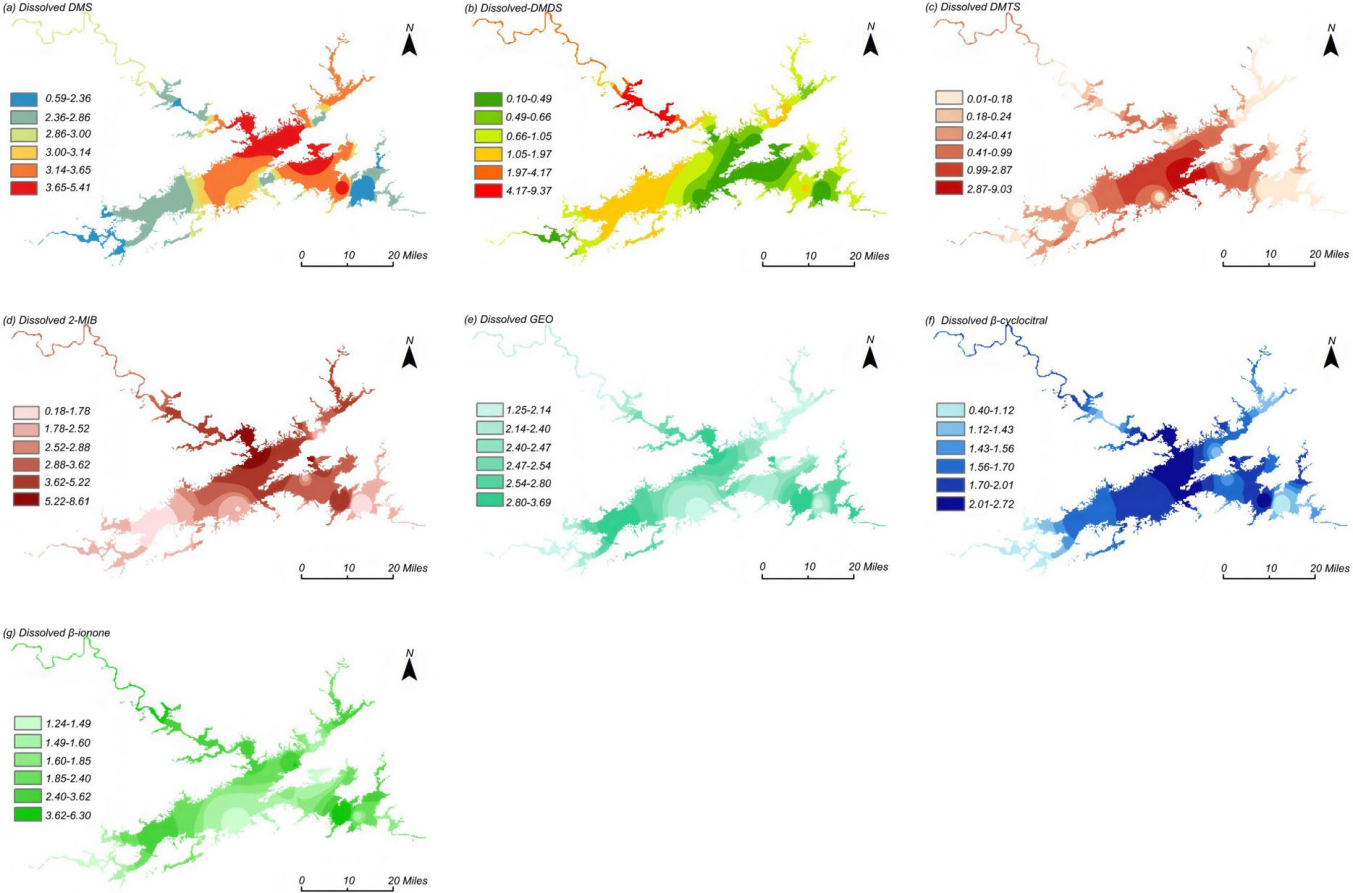
Spatial distribution characteristics of dissolved of odor compounds in Qiandaohu Reservoir, DMS (**a**); DMDS (**b**); DMTS (**c**); 2-MIB (**d**); GEO (**e**); β-cyclocitral (**f**); β-ionone (g) (ng/L)

In eutrophic water, the input of high concentrations of nutrients and the falsification of value-added will promote the release of DMS, and its decomposition products are considered to be an important reason for the odor of eutrophic water and may affect the safety of drinking water, such as DMDS and DMTS (Yu et al., 2019). The lake-wide average concentration of particulate DMS (p-DMS) is 0.20 ng/L, with a variation range of 0.008 to 0.36 ng/L; the lake-wide average concentration of dissolved DMS (d-DMS) is 3.05 ng/L, with a variation range of 0.59 to 5.42 ng/L (Fig. 1a). The spatial distribution of p-DMS concentrations generally follows the trend: southwest > northeast > southeast > northwest > central lake areas. As shown in Fig. 2a, the spatial distribution of d-DMS concentrations shows a different pattern: central lake area > southeast > northeast > southwest > northwest lake areas, with all absolute concentrations being below the odor threshold and posing no safety risk. The highest concentration of DMS is observed at Maotoujian, while lower levels are detected at Jiekou, potentially due to the low boiling point of DMS (38 °C) and the higher water flow velocity at Jiekou, causing volatilization of DMS \compounds, resulting in lower detected concentrations.

The lake-wide average concentration of particulate DMDS (p-DMDS) is 0.27 ng/L, with a variation range of 0.05 to 1.38 ng/L; the lake-wide average concentration of dissolved DMDS (d-DMDS) is 1.17 ng/L, with a variation range of 0.10 to 9.38 ng/L (Fig. 1b). The spatial distribution of p-DMDS concentrations generally follows the trend: northeast > southeast > northwest > southwest > central lake areas. As shown in Fig. 2b, the spatial distribution of d-DMDS concentrations exhibits a different pattern: northwest > northeast > southwest > southeast > central lake areas, but with relatively low absolute concentrations. The highest concentrations of p-DMDS are found at Hangtou Island and Mishan, while higher concentrations of d-DMDS are observed at Jiekou and Weiping Forest Farm, likely related to the shallower water depth and higher nutrient levels conducive to algal growth at these locations.

The lake-wide average concentration of particulate DMTS (p-DMTS) is 0.15 ng/L, with a variation range of 0.018 to 1.31 ng/L; the lake-wide average concentration of dissolved DMTS (d-DMTS) is 0.65 ng/L, with a variation range of 0.01 to 9.03 ng/L (Fig. 1c). The spatial distribution of p-DMTS concentrations generally follows the trend: central lake area > northeast > southeast > northwest > southwest lake areas. As shown in Fig. 2c, the spatial distribution of d-DMTS concentrations shows a different pattern: central lake area > northeast > southwest > northwest > southeast lake areas, with all absolute concentrations being below the odor threshold and posing no safety risk. Spatially, p-DMTS peaks at Hangtou Island and Chengzhong Lake (algae-rich zones), while d-DMTS dominates at Santiandi Island (high-DOC inflow)—reflecting colloidal vs. DOM-driven distributions. All values remain below odor threshold.

#### Spatial distribution characteristics of 2-MIB and GEO

2-MIB and GEO usually coexist in eutrophic freshwater systems and can be produced by similar algal communities through biological metabolisms, such as cyanobacteria or actinomycetes (van der Ploeg et al., 1992). In addition, previous studies have shown that both are the main sources of earthy odor in drinking water and reservoirs and can be perceived by human olfaction at extremely low concentrations (Li et al., 2018). Therefore, it is important to detect the changes of 2-MIB and GEO concentrations in the water environment to indicate the degree of eutrophication of lakes.

The lake-wide average concentration of particulate 2-MIB (p-2-MIB) is 0.31 ng/L, with a variation range of 0.06 to 10.2 ng/L; the lake-wide average concentration of dissolved 2-MIB (d-2-MIB) is 3.19 ng/L, with a variation range of 0.19 to 8.61 ng/L (Fig. 1d). The spatial distribution of p-2-MIB concentrations generally follows the trend: northwest > central > northeast > southeast > southwest lake areas. As shown in Fig. 2d, the spatial distribution of d-2-MIB concentrations shows a similar pattern: central > northwest > northeast > southeast > southwest lake areas, with all absolute concentrations being below the odor threshold and posing no safety risk. Higher concentrations of d-2-MIB are observed at Xiaojinshan, while the highest concentrations of p-2-MIB are found at Weiping Forest Farm, Xiaojinshan, Chengzhōng Lake.

The lake-wide average concentration of particulate GEO (p-GEO) is 0.16 ng/L, with a variation range of 0.02 to 0.35 ng/L; the lake-wide average concentration of dissolved GEO (d-GEO) is 0.59 ng/L, with a variation range of 0.19 to 1.03 ng/L (Fig. 1e). The spatial distribution of p-GEO concentrations generally follows the trend: central > northwest > southeast > northeast > southwest lake areas. As shown in Fig. 2e, the spatial distribution of d-GEO concentrations shows a different pattern: northwest > southeast > southwest > central > northeast lake areas. Higher concentrations of d-GEO are observed at Xiaojinshan, Maotoujian, Jinzhupai, and County Water Plant, but with very low absolute concentrations, all below the odor threshold and posing no safety risk. The highest concentrations of p-GEO are found at Xiaojinshan, Santiandi Island, and Chengzhong Lake.

#### Spatial distribution characteristics of β-cyclocitral and β-ionone

In eutrophic water, β-cyclocitral and β-ionone are two common volatile organic compounds, which are produced by the oxidative breakdown of β-carotene within algae, primarily originating from spherical blue-green algae (Zhang et al., 2013; Zorn et al., 2003). In some detection systems, they can be used as important indicator compounds for water quality detection and ecological health assessment, which can identify the trend of eutrophication in advance, so as to provide a reference for water quality protection and ecological restoration.

The lake-wide average concentration of particulate β-cyclocitral (p-β-cyclocitral) is 2.28 ng/L, with a variation range of 1.25 to 3.70 ng/L; the lake-wide average concentration of dissolved β-cyclocitral (d-β-cyclocitral) is 1.63 ng/L, with a variation range of 0.41 to 2.72 ng/L (Fig. 1f). The spatial distribution of p-β-cyclocitral concentrations generally follows the trend: southwest > northwest > central > northeast > southeast lake areas. As shown in Fig. 2f, the spatial distribution of d-β-cyclocitral concentrations shows a different pattern: northwest > central > southeast > northeast > southwest lake areas; higher concentrations of d-β-cyclocitral are observed at Xiaojinshan, Pailing Water Plant, and Santiandi Island, but with relatively low absolute concentrations, all below the odor threshold and posing no safety risk. The highest concentrations of p-β-cyclocitral are found at Xiaojinshan, Chengzhong Lake, Jinzhupai, and Santiandi Island.

The lake-wide average concentration of particulate β-ionone (p-β-ionone) is 1.99 ng/L, with a variation range of 1.24 to 6.30 ng/L; the lake-wide average concentration of dissolved β-ionone (d-β-ionone) is 0.33 ng/L, with a variation range of 0.05 to 0.74 ng/L (Fig. 1g). The spatial distribution of p-β-ionone concentrations generally follows the trend: northwest > central > southwest > northeast > southwest lake areas. As shown in Fig. 2g, the spatial distribution of d-β-ionone concentrations shows a different pattern: northwest > southeast > northeast > southwest > central lake areas. Higher concentrations of d-β-ionone are observed near Xiaojinshan, Maotoujian, Hangtou Island, and County Water Plant, but with relatively low absolute concentrations, all below the odor threshold and posing no safety risk. The highest concentrations of p-β-ionone are found at Xiaojinshan, Weiping Forest Farm, and Jiekou.

In general, in the horizontal direction, the concentration distribution of these odor compounds is different. The main algal-derived odor compounds 2-MIB and GEO show higher concentration values in northwest, southwest, and northeast lake regions during high-temperature seasons. Locations where concentrations exceed the odor threshold include Jie Kou, Xiao Jinshan, Weiping Forest Farm, Mao Toujian, Bai Mufan, and Hang Tou Island. These sites are close to the shore or near bays, where slow water flow, nutrient-rich inflow, and favorable light and temperature conditions are conducive to extensive algal proliferation and accumulation, leading to higher concentrations of odor compounds (Chen et al., 2010).

### Vertical distribution characteristics of odor compounds in Qiandaohu Reservoir

#### Vertical distribution characteristics of DMS, DMDS, and DMTS

Monthly monitoring data from Qiandaohu Reservoir indicate that the vertical gradient variations for particulate DMS, DMDS, and DMTS range from 0.003 to 1.46 ng/L, 0.003 to 3.28 ng/L, and 0.003 to 0.95 ng/L, respectively. The maximum value for particulate DMS was observed at a depth of 3 m below the surface in April 2024, while the maximum values for particulate DMDS were recorded at depths of 10 m in July 2023. Additionally, particulate DMTS reached its peak at a depth of 5 m in March 2024, surpassing its odor threshold. For dissolved DMS, DMDS, and DMTS, the variation ranges were 0.48–11.99 ng/L; 0–9.34 ng/L; and 0–71.70 ng/L, with their maxima occurring at depths of 5 m in September 2023, 20 m in July 2023, and 1 m in September 2023, respectively. Overall, the vertical gradient changes for odor compounds DMS, DMDS, and DMTS are minimal during autumn and winter but exhibit an increasing then decreasing trend during spring and summer. At depths exceeding 10 m, these odor compounds rapidly decrease, resulting in lower absolute concentrations of the dissolved state.

#### Vertical distribution characteristics of 2-MIB and GEO

**Fig. 3:**
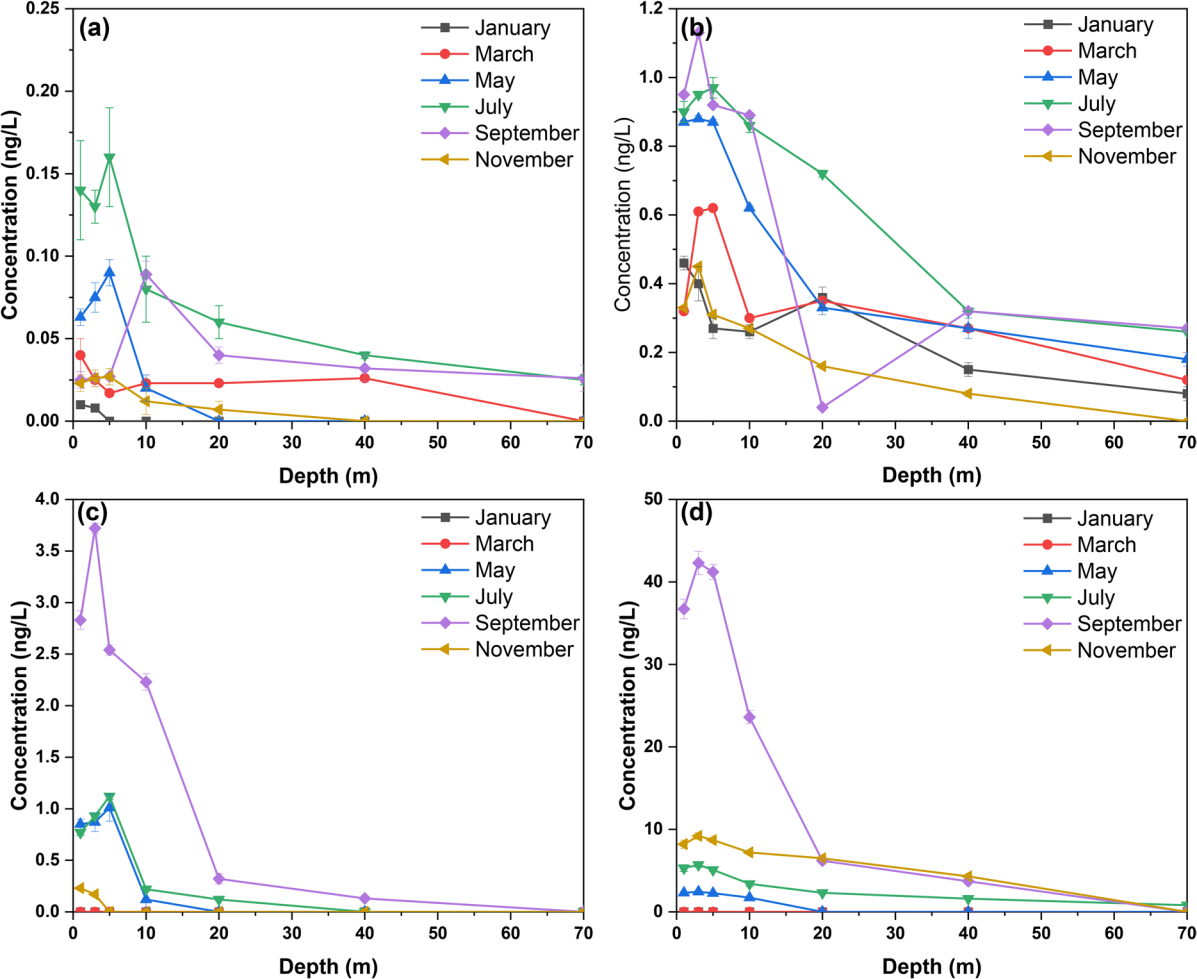
Spatial distribution of major odor compounds GEO and 2-MIB in Qiandaohu Reservoir. Particulate-GEO (**a**); dissolved-GEO (**b**); particulate-2-MIB (**c**); dissolved-2-MIB (**d**)

As shown in Fig. 3, the vertical gradient variations for particulate 2-MIB and GEO range from 0–6.31 ng/L and 0–0.57 ng/L, respectively. The maximum values for particulate 2-MIB and GEO were observed at depths of 5 m in September 2023 and July 2023. For dissolved 2-MIB and GEO, the variation ranges were 0.16–65.94 ng/L and 0–1.23 ng/L, with their maxima also noted at depths of 5 m in September 2023. Overall, the vertical gradient changes for odor compounds 2-MIB and GEO are minimal during autumn and winter but show an increasing and then decreasing trend during spring and summer. At depths exceeding 10 m, these odor compounds rapidly decrease, resulting in lower absolute concentrations of the dissolved state. In September, the concentrations of 2-MIB and GEO exceeded their respective odor thresholds by factors ranging from one to six times.

#### Vertical distribution characteristics of β-cyclocitral and β-ionone

Monthly monitoring data indicate that the vertical gradient variations for particulate β-cyclocitral and β-ionone range from 0.08 to 15.78 ng/L and 0 to 5.57 ng/L, respectively. The maximum values for particulate β-cyclocitral and β-ionone were observed at depths of 5 m in July 2023. For dissolved β-cyclocitral and β-ionone, the variation ranges were 0–7.05 ng/L and 0–27.15 ng/L, with their maxima recorded at depths of 40 m in January 2024 and at the surface layer in September 2023, respectively. Overall, the vertical gradient changes for odor compounds β-cyclocitral and β-ionone are minimal during autumn and winter but exhibit an increasing then decreasing trend during spring and summer. The dissolved state concentrations remain relatively low; however, β-ionone exceeded its odor threshold by factors ranging from one to four times in September.

Vertically, influenced by factors such as light intensity, temperature, sediments, hydrological conditions, and dissolved oxygen, the distribution of odor compounds is not uniform across the surface, middle, and bottom layers of the water body. Overall, the concentration of odorous substances increases and reaches a peak from 0 to 5 m, and then decreases rapidly when the depth exceeds 10 m. This may be due to the fact that most algae reproduce at the surface of the lake so the odor compounds produced by their metabolism mainly remain in the submersible area.

### Variation in phytoplankton community structure in Qiandaohu Reservoir

**Fig. 4:**
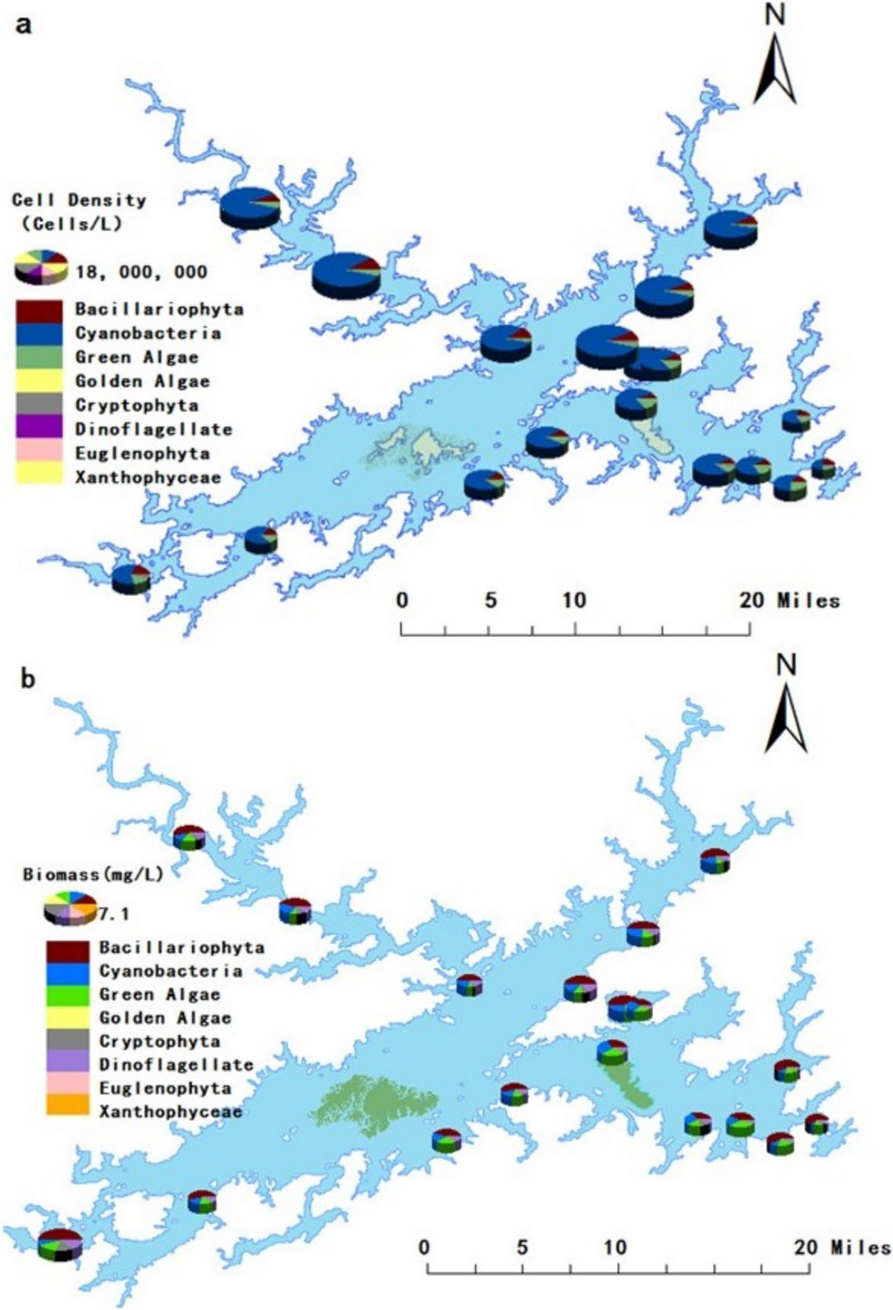
Spatial distribution maps of phytoplankton cell density (**a**) and biomass composition (**b**) in Qiandaohu Reservoir

Although the formation mechanism is basically the same, the composition and dominant genera of algae communities in eutrophic water bodies may differ due to regional climate and water quality variations, ultimately affecting the composition and content of odor compounds (Su et al., 2021). During the monthly survey, a total of 8 phyla, 92 genera, and 186 species of phytoplankton were identified. These included Cyanobacteria (18 genera, 31 species), Chlorophyta (43 genera, 93 species), Bacillariophyta (21 genera, 47 species), Cryptophyta (2 genera, 4 species), Chrysophyceae (2 genera, 3 species), Pyrrhophyta (4 genera, 4 species), Euglenophyta (1 genus, 3 species), and Xanthophyta (1 genus, 1 species). Chlorophyta had the highest species richness (50.0%), followed by Bacillariophyta (25.3%) and Cyanobacteria (17.7%). The proportions of Cryptophyta, Xanthophyta, Pyrrhophyta, and Chrysophyceae were each below 5%. Dominant cyanobacterial genera in Qiandao Lake Reservoir included *Pseudoanabaena*, *Leptolyngbya*, *Microcystis*, *Planktothrix*, *Dolichospermum*, and *Oscillatoria*, which contributed significantly to cell density. As shown in Fig. 4a, algal density followed: northwest > central > northeast > southwest > southeast lake regions, with Cyanobacteria dominating all areas. The variation range of phytoplankton cell density was 1.27 × 10^5^–5.03 × 10^7^ cells/L, with an average of 1.33 × 10^7^ cells/L. As illustrated in Fig. 4b, the biomass of algae showed a pattern of northwest lake area > southwest lake area > northeast lake area > central lake area > southeast lake area. The variation range of phytoplankton biomass was 0.10–5.62 mg/L, with an average of 2.00 mg/L, mainly dominated by Bacillariophyta biomass. The proportion of Cyanophyta biomass increased from July to September, with the maximum biomass occurring in May 2022, May 2023, and August 2023, and the minimum in January and February 2024. Interestingly, the seasonal changes and regional differences of algae biomass in Qiandao Lake are basically consistent with the seasonal changes and distribution characteristics of odor compounds, indicating that odor compounds are derived from the reproduction and decline of algae.

### Temporal and spatial variations of enzyme synthesis in Qiandaohu Reservoir

Extensive research confirms that algae, particularly cyanobacteria, chrysophytes, diatoms, and chlorophytes, constitute primary sources of odor pollution in aquatic ecosystems (Srinivasan & Sorial, 2011). Cyanobacteria are frequently implicated as dominant producers of taste-and-odor compounds, aligning with our detection of GEO and 2-MIB as key drivers of off-flavors in Qiandao Lake. To identify priority control zones, we quantified the spatiotemporal distribution of geo and mib synthase genes across the lake from May 2023 to April 2024.

**Fig. 5:**
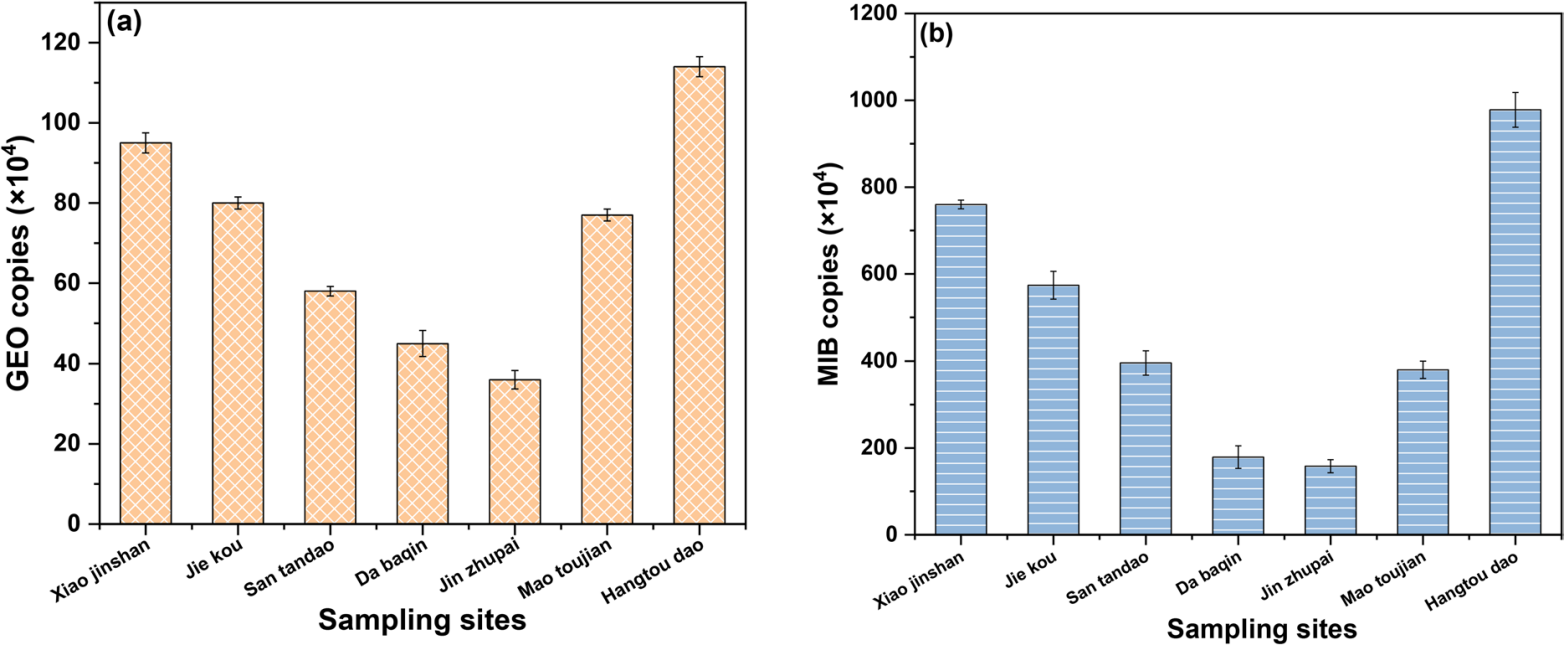
Spatial distribution of GEO (**a**) and MIB (**b**) synthases across various lake regions in Qiandaohu Reservoir

GEO synthase genes exhibited consistently low abundance (Fig. 5), peaking modestly from May to September before declining in cooler months. In contrast, MIB synthase genes were ubiquitously detected at higher concentrations, with pronounced peaks from June to October. Spatially, the lowest annual GEO gene abundance occurred near Qiandaohu Dam and the water diversion project, whereas elevated mib gene copies clustered around Hangtou Island, Xiaojin Mountain, and Jiekou. Critically, our 16S rDNA analysis revealed that *Planktothrix agardhii* and *Microcystis spp.* dominated cyanobacterial communities at high-odor sites, corroborating findings from Gonghu Bay where 2-MIB correlated solely with *P. agardhii* density during thermal stratification (Ren et al., 2024). Expanding this linkage, mcyB gene quantification demonstrated that toxigenic strains of Microcystis and P. agardhii co-occurred with odor peaks. Correlation analyses further indicated: (1) Dissolved 2-MIB strongly associated with Microcystis*/P. agardhii* cell density/biomass (*ρ* > 0.82, *p* < 0.01); (2) Particulate 2-MIB correlated exclusively with *P. agardhii* metrics (*ρ* > 0.91, *p* < 0.001). This mechanistic evidence, integrating functional gene (MIB, mcyB) and community (16S rDNA) data, establishes *P. agardhii* as the paramount 2-MIB producer in Qiandao Lake. Consequently, controlling this species’ proliferation represents the most targeted strategy for mitigating odor outbreaks, particularly in high-risk zones identified by gene hotspots.

### Relationship between key odor compounds and environmental factors in Qiandaohu Reservoir

#### Correlation analysis of key odor compounds and environmental factors

Generally, environmental factors such as water temperature, dissolved oxygen, pH, nutrient salts, and organic matter concentrations can influence the growth and reproduction of planktonic algae, thereby affecting the concentration and distribution of algal-derived odor compounds. However, the environmental factors influencing algal-derived odor compounds vary among different studies. For instance, investigations into odor compounds in Taihu Lake found that total nitrogen, COD _Mn_, dissolved oxygen, pH, and temperature not only affect the production of odor compounds in water bodies but also have an impact on their decomposition and transformation. At the same time, reports have also pointed out that nitrogen content affects the release of certain odor compounds (Olsen et al., 2016). Moreover, in the previous study, DMDS and DMTS were significantly correlated with TN (Yu et al., 2019). Considering that odor compounds are closely related to local climate and water environment conditions, the influencing factors vary from region to region. Therefore, monthly sampling data were used to perform correlation analyses between environmental indicators and odor compounds at all monitoring sites, with the expectation of understanding and controlling the key factors affecting the odor emitted from Qiandaohu Reservoir.

**Fig. 6:**
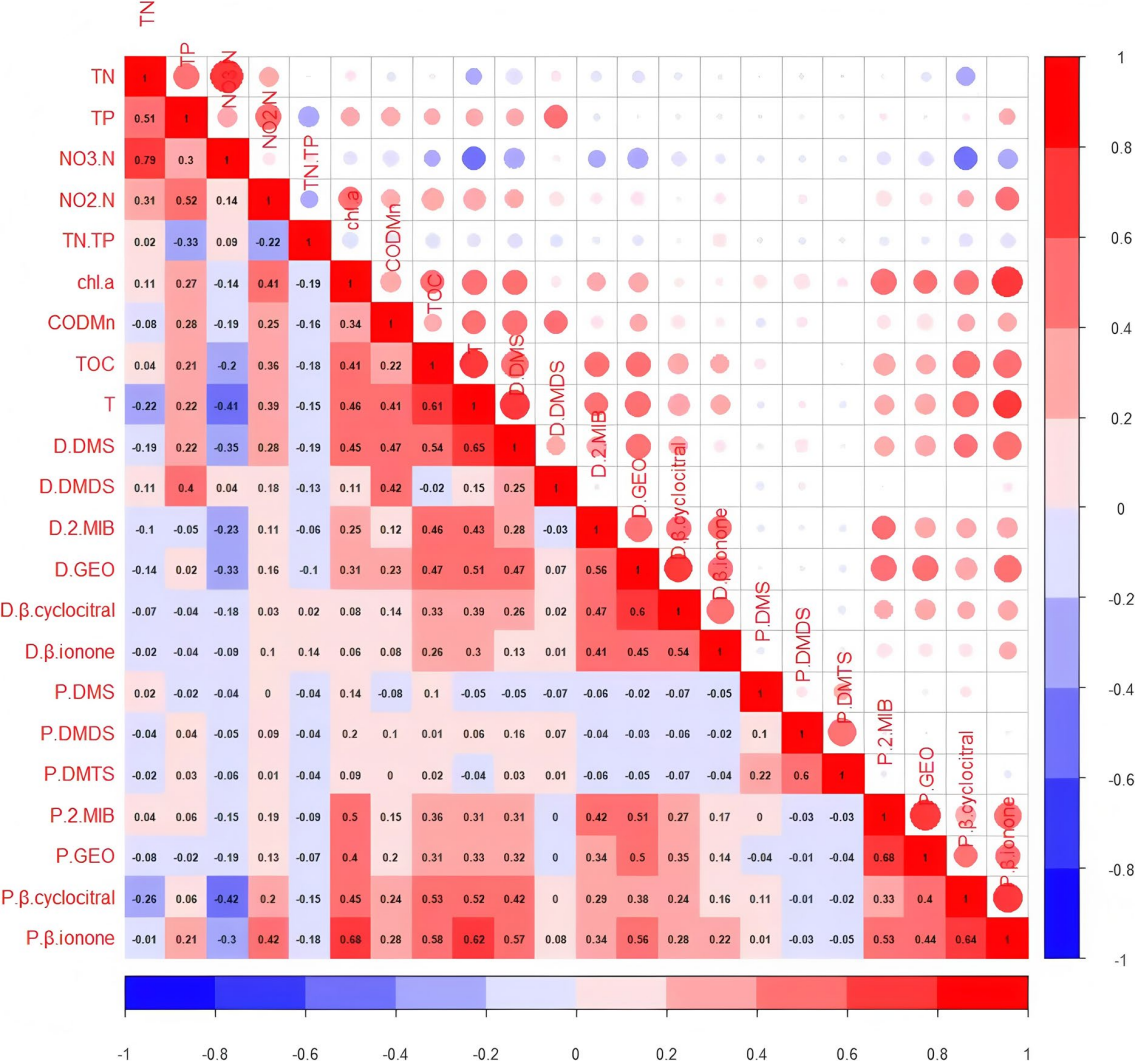
Heatmap showing correlation coefficients between environmental parameters and odor compounds in Qiandao Lake. Blue hues indicate negative correlations; red hues indicate positive correlations

The significant positive correlation between dissolved DMS and Chl-a observed in our study (Fig. 6) aligns with findings from Xu et al. (2025) in Lake Qiandaohu, where a near 1:1 linear relationship between Chl-a and TP was reported, indicating that TP enrichment directly stimulates algal biomass (Chl-a). Similarly, the positive correlation between DMDS and TP (*r* = 0.40) in our results further supports the role of phosphorus in promoting phytoplankton productivity, as higher TP levels likely enhance algal-derived organic precursors for DMDS production. The negative correlation between DMS and NO₃-N (r =  − 0.35) suggests potential N-limitation or shifts in phytoplankton community composition under high N:P ratios, consistent with Xu et al. (2025), who demonstrated that P-limitation dominates in deep subtropical reservoirs like Qiandaohu, while N co-limitation occurs only in localized riverine zones with low N:P ratios. The strong positive relationship between DMS/DMDS and COD_Mn_ (*r* = 0.40–0.42) implies that elevated organic matter (a proxy for algal-derived organics) fuels microbial DMS/DMDS production, reinforcing the link between nutrient-driven eutrophication and sulfur metabolite dynamics. Collectively, these results underscore TP as a critical driver of both Chl-a and sulfur metabolites (e.g., DMS/DMDS), advocating for P-focused management strategies to mitigate eutrophication and associated biogeochemical impacts in subtropical reservoirs.

Both dissolved 2-MIB and dissolved GEO exhibited significant positive correlations with total organic carbon (TOC) and temperature, but significant negative correlations with NO_3_-N (− 0.23; − 0.33), which was consistent with previous research. Low concentrations of nitrate nitrogen facilitate the synthesis of GEO, but as nitrate-nitrogen concentration increases, the synthesis of GEO is inhibited (Saadoun et al., 2001). Similarly, dissolved β-cyclocitral and dissolved β-ionone showed significant positive correlations with TOC and temperature. Dissolved odor compounds exhibit a low correlation with environmental factors, which may be attributed to microbial degradation, degradation caused by pH and light, adsorption by particulates, and volatilization due to disturbances in lake water bodies.

The particulate forms of DMS, DMDS, and DMTS showed lower correlations with various environmental factors, indicating less influence from these factors. Conversely, particulate 2-MIB and particulate GEO exhibited significant positive correlations with Chl a, COD _Mn_, TOC, and temperature. Particulate β-cyclocitral showed significant positive correlations with TN, NO_3_-N, NO_2_-N, Chl a, COD _Mn_, TOC, and temperature, but a significant negative correlation with NO_3_-N (− 0.43). Particulate β-ionone demonstrated significant positive correlations with TP, NO_2_-N, TN/TP, Chl a, COD _Mn_, TOC, and temperature, and a significant negative correlation with NO_3_-N (− 0.30). It is evident that non-biological factors and other environmental conditions can significantly influence the content of odor compounds such as dimethyl isoborneol, GEO, DMS, and DMTS in water bodies. The results of this study show that odor compounds in Qiandaohu Reservoir are greatly influenced by TN, TP, and TN/TP, with TP, COD _Mn_, TOC, and temperature all being environmental factors affecting the concentration of odor compounds.

#### Relationship between odor compounds and algae in Qiandaohu Reservoir

The relationship between odor compounds and algae in water bodies is a popular research direction at home and abroad. Monthly sampling data were analyzed to investigate the correlations between odor compounds and the biomass and cell density of dominant phytoplankton species at all monitoring sites. The results revealed significant positive correlations between dissolved DMS and the biomass and cell density of cyanobacteria, including Microcystis spp., Planktothrix spp., Anabaena spp., and Oscillatoria spp. However, dissolved DMDS showed weak correlations with the selected algal species. Meanwhile, among the odor compounds exceeding the odor threshold, dissolved state −2-MIB shows a strong correlation with the cell density and biomass of Planktothrix spp; Dissolved state -GEO is closely related to the cell density and biomass of Anabaena, and β-ionone has a strong correlation with the biomass and cell density of blue-green algae and Planktothrix spp, which was consistent with previous research (Smith et al., 2008). Dominant Microcystis in blue-green algal blooms, along with accompanying large growth of Oscillatoria, Anabaena, Phormidium, and Planktothrix, are the main sources of β-ionone and 2-MIB. In addition, in eutrophic lakes, the increase in algal biomass usually leads to the accumulation of odorous compounds, especially when algae decay. In our study, we found that particulate 2-MIB showed a strong correlation with the cell density and biomass of Planktothrix spp. (*r*^2^ = 0.4). Particulate GEO was closely related to the cell density and biomass of Oscillatoria spp. (0.44). Particulate β-cyclocitral exhibited a strong correlation with the biomass of cyanobacteria, while particulate β-ionone showed strong correlations with the biomass and cell density of cyanobacteria and Planktothrix spp. (0.42). Therefore, in order to reduce the adverse effects of algal secondary metabolites on the surrounding environment, appropriate artificial interventions can be carried out during the hot season, including strengthening upstream water quality control, increasing the flow of bays and tributaries, or introducing specific plankton to control the population of target algae.

## Conclusion

(1) The concentrations of DMS, DMDS, and β-cyclocitral in Qiandaohu Reservoir were all below their respective odor thresholds; however, a certain proportion of samples exceeded the odor threshold for DMTS, 2-MIB, GEO, and β-ionone. The primary odor compounds in Qiandaohu Reservoir are 2-MIB and GEO, which produce earthy and musty odors, respectively. On average, the concentrations of these odor compounds in Qiandaohu Reservoir were relatively low, with lower levels observed in autumn and winter, and relatively higher concentrations in spring and summer;

(2) The annual average relative abundance of enzymes involved in the synthesis of GEO and MIB in Qiandaohu Reservoir was relatively low, but it was higher in some bay areas during the summer, indicating a potential risk of odor pollution;

(3) The presence of odor compounds in Qiandaohu Reservoir was significantly influenced by TN, TP, and the TN/TP ratio. Several odor compounds exhibited a significant negative correlation with NO_3_-N, while temperature, TOC, and COD_Mn_ were key environmental factors affecting the presence of these compounds;

(4) Cyanobacteria were identified as the primary biological source of odor compounds in Qiandaohu Reservoir, particularly filamentous cyanobacteria such as *Planktothrix agardhii* and *Anabaena spp.*

## Supplementary Information

Below is the link to the electronic supplementary material.

Supplementary file1 (DOCX 266 KB)

## Data Availability

No datasets were generated or analysed during the current study.
