# Supplementary material for: Temporal and spatial pattern, sources, and main controlling factors of odor compounds in Qiandaohu Reservoir
Source: Environ Monit Assess. 2025 Aug 20;197(9):1031. doi: 10.1007/s10661-025-14505-5 (PMC12367828; doi:10.1007/s10661-025-14505-5)

**Supporting Information for**

**Temporal and Spatial pattern, sources, and main controlling factors of odor compounds in Qiandaohu Reservoir**

Yi Chi^1^, Zhanpu Tang^2^, Yingjun Zhu^1^, Lingjia Wang^1^, Tianli Chen^3^, Ping He^1^, Yuxin Chen^1^, Tong Sun^4^, Siying Cai^4,*^, Weijun zhang^4^

**^1^** Hangzhou Ecological and Environmental Monitoring Center of Zhejiang Province, Hangzhou 310003, Zhejiang, China.

**^2^** Quality testing center for architectural engineering of Zhejiang province Co. Ltd., Hangzhou 310012, Zhejiang, China.

**^3^** School of Water Resources and Environmental Engineering, East China University of Technology, Shanghai 200237, China.

**^4^** School of Environmental Studies, China University of Geosciences, Wuhan 430074, Hubei, China.

**Text S1 Specific information on the sampling sites.**

In this study, a total of 17 sampling sites were selected in the whole Qiandao Lake, which were located in the southeast, northeast, southwest and northwest directions of the lake, and the central area of the lake was also set up sampling sites. **Figure S1** shows the detailed location of each sampling site. The names of the sampling points are as follows: S1 (Jie Kou), S2 (Xiao Jinshan), S3 (San Tanda), S4 (Da baqian), S5 (Hang toudao), S6 (Mao toujian), S7 (Jin zhupai), S8 (Weiping Forest Farm), S9 (Mao zhuyuan), S10 (Laoshan Outlet), S11 (Bai mufan), S12 (Pailing Waterworks), S13 (Mi shan), S14 (Xi yuan), S15 (Chengzhong Lake), S16 (County Waterworks), and S17 (Xuyuan Forest Farm).

**Text S2 The detailed process of stratified sampling.**

Stratified sampling was carried out at 6 points of the national-controlled sections (Jie Kou, Xiao Jinshan, San Tandao, in front of the Dam, Hang Toudao, and Mao Tou jian) and 1 point of the water diversion project (Jin Zhupai). For the national-controlled sections, the stratified sampling rule was to conduct intensive sampling within 20 meters underwater (at depths of 1 meter, 3 meters, 5 meters, 10 meters, and 20 meters). For the sampling points with a water depth of 40 meters, an additional sample was taken at 40 meters, and for those with a water depth of 70 meters, additional samples were taken at 40 meters and 70 meters. For the sampling point of the water diversion project (Jin zhupai), water samples were collected at depths of 1 meter, 10 meters, and 30 meters underwater. For the remaining 10 sampling points (Weiping Forest Farm, Mao zhuyuan, Lao Shan Outlet, Mishan, Xi yuan, Chengzhong Lake, County Waterworks, Bai Mufan, Xu yuan Forest Farm, and Pailing Waterworks), water samples were collected at a depth of 1 meter underwater. The water samples at different depths were collected by using a specific collector and then transported to the laboratory for subsequent analysis.

**Table S1 Details of primers used in this study.**

| **Target genes** | **Sequence (5'-3')** | **Types** | **PCR Products(bp)** |
| --- | --- | --- | --- |
| CYA | CGGACGGGTGAGTAACGCGTGA | Forward Primer | 1369 |
|  | TACGGYTACCTTGTTACGACTT | Reverse Primer |  |
| 16S rDNA | ATGTGCCGCGAGGTGAAACCTAAT | Forward Primer | 202 |
|  | TTACAATCCAAAGACCTTCCTCCC | Reverse Primer |  |
| GEO | TGGTATGTNTGGGTRTTCTT | Forward Primer | 311 |
|  | ATGTATTCRATGGGGTTRGC | Reverse Primer |  |
| MIB | CGACAGCTTCTACAYCYCCATGAC | Forward Primer | 200 |
|  | CGCCGCAATCTGTAGCACCAT | Reverse Primer |  |
| mcy*B* | CCTACCGAGCGCTTGGG | Forward Primer | 78 |
|  | GAAAATCCCCTAAAGATTCCTGAGT | Reverse Primer |  |

**Figure S1** Sampling sites of Qian daohu Reservoir.


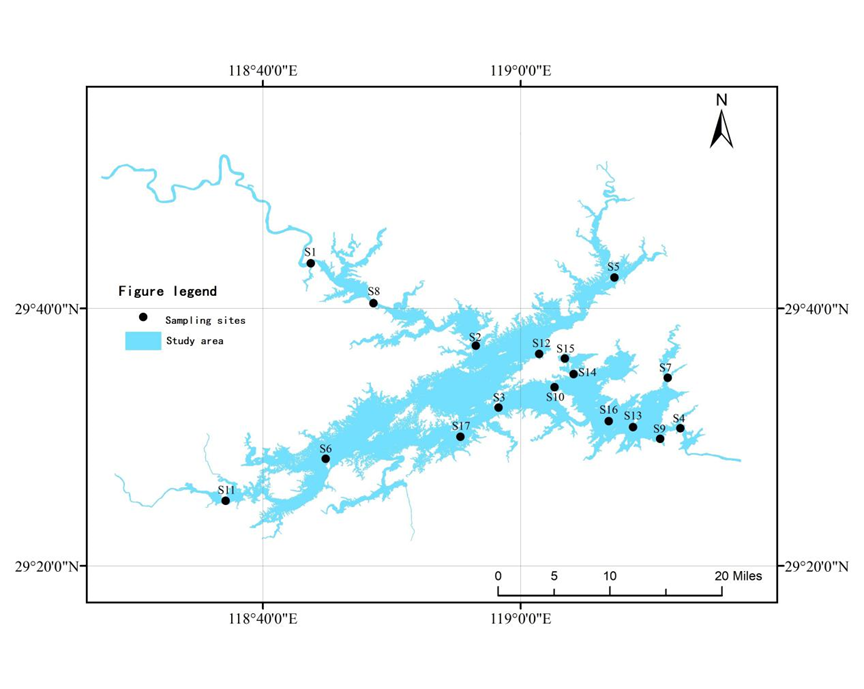

Supplement: Supplementary file 1 — Supplementary file1 (DOCX 266 KB) [file 10661_2025_14505_MOESM1_ESM.docx]
